# Impact of fat content of oral nutritional intake on visibility and diameter of the thoracic duct: a prospective study

**DOI:** 10.1038/s41598-026-62725-7

**Published:** 2026-07-21

**Authors:** Judith Padberg, Andreas Henkel, Alois Martin Sprinkart, Jennifer Nadal, Alexander Isaak, Julian A. Luetkens, Christopher Hart, Claus C. Pieper

**Affiliations:** 1 Division for Minimally-invasive Lymph Vessel Therapy, Department of Diagnostic and Interventional Radiology, University Hospital Bonn, Venusberg-Campus 1, 53127 Bonn, Germany; 2 Department of Medical Biometry, Informatics and Epidemiology, University Hospital Bonn, Bonn, Germany; 3 Department of Pediatric Cardiology, Children’s Hospital, University Hospital of Bonn, Bonn, Germany

**Keywords:** Lymphatic imaging, Thoracic duct, Lymphangiography, MRI, Chylothorax, Diseases, Endocrinology, Health care, Medical research

## Abstract

The aim of this study was to investigate visibility and diameter changes of the thoracic duct (TD) on non-contrast MR-lymphangiography (MRL) after oral nutritional intake with different fat-content. Healthy adults prospectively underwent non-contrast thoracic MRL at 1.5 T on three separate days following an overnight fasting period. After baseline MRL, patients received standardized meals: (1) high-fat, (2) medium-chain-triglyceride (MCT), (3) fat-free. Thereafter hourly MR-scans were obtained for 6 h. TD-visibility was evaluated for the superior and inferior thoracic and abdominal TD-segment on a 6-point-scale (1:best; 6:worst). TD-diameters were measured independently by two readers. Statistical analysis was performed to evaluate systematic differences between the different meals using paired t-tests and a linear mixed-model analysis. 154 MRI-scans were performed in 22 volunteers (16 female, mean age 29.7 ± 14.3 years). Overall TD-visibility improved significantly after the high-fat meal (baseline: 2.9 ± 0.9 vs. 6 h-examination: 2.4 ± 0.8; *p* < 0.001), while it slightly deteriorated after the MCT-meal (2.7 ± 0.9 vs. 3.2 ± 1.0; *p* < 0.001) and fat-free meal (2.8 ± 0.9 vs. 3.1 ± 1.0; *p* = 0.006). TD-diameter increased after the high-fat meal with a maximum at 5 h postprandially in the abdominal (2.4 ± 0.6 mm vs. 2.9 ± 1.0 mm, + 21.5%, *p* < 0.001) and lower thoracic segment (2.1 ± 0.4 mm vs. 2.5 ± 0.5 mm, + 16.8%, *p* < 0.001) while the maximum mean increase in the upper thoracic segment was observed at 2 h postprandially (2.0 ± 0.3 mm vs. 2.3 ± 0.5 mm, + 13.8%). In contrast, no significant increase in TD-diameters over time was observed for the fat-free and MCT-meals. The observed improvement in TD visibility and increase in TD diameter after a high-fat meal suggest a relation to lymphatic resorption of long-chain triglycerides. Conversely, this effect was not observed after an MCT- and fat-free meal.

## Introduction

The lymphatic vascular system plays a major role in fluid homeostasis of the human body, returning tissue fluid to the venous circulation^1,2^. Intestinal lymph vessels additionally transport resorbed dietary fatty acids. All lymph vessels of the lower body converge in the upper retroperitoneum to form the cisterna chyli which drains through the thoracic duct (TD) – the largest lymph vessel in the human body. The TD runs from the upper abdomen alongside the thoracic aorta ultimately draining into the venous system through the left lymphatic-venous junction^3–5^. The exact mode of dietary fat resorption is not yet fully understood, but depends on the molecular size of fatty acids^6^. While long-chain fatty acids (> 12 carbon atoms) are transported in the form of chylomicrons by the lymphatic system (the triglyceride rich lymphatic fluid is termed chyle), short and medium-chain triglycerides (< 12 carbon atoms) are absorbed directly into the portal venous circulation^7–10^. Lymph-flow in the intestinal lymph vessels and by extension in the TD is therefore thought to be depended on diet with increasing flow after a fat-rich meal^4,5,7,11,12^.

This supposed mechanism is exploited in the treatment of central lymphatic (i.e. chylous) leakages (e.g. chylothorax). Parenteral nutrition, fat-free or medium-chain triglyceride diets are employed in conservative management of these chylous leakages in an effort to reduce central lymph flow to facilitate spontaneous healing of the leakage^2,13–15^. In cases refractory to conservative therapy, lymphatic imaging has been increasingly employed in recent years^16,17^. Especially MR-Lymphangiography (MRL) is particularly useful in evaluating the central lymphatic system. So called non-contrast MRL employs heavily T2-weighted imaging and can depict lymphatic anatomy non-invasively^5,18–26^. Recent studies have suggested that a high-fat meal prior to non-contrast MRL can improve TD-visibility compared to the fasting state^4,5,11^, inferring that this is due to an increased TD-diameter because of higher lymph flow after the high-fat meal. However, quantitative assessment of diameter changes depending on diet are scarce^11^. Furthermore, the effects of an MCT-diet on TD-diameter have so far not been investigated. This is of particular interest as an MCT-diet is typically prescribed in the treatment of chylous effusions.

The aim of the present study was therefore to investigate qualitative visualization rates on non-contrast MRL as well as quantitative diameter changes of the thoracic duct after meals with different fat content.

## Materials and methods

### Subject inclusion

Adult healthy volunteers (age ≥ 18 years) without disorders affecting the central lymphatic load (e.g. kidney or liver disease) as well as without signs of lymphatic disease could participate in the study. The study was approved by the local institutional review board of the Medical Faculty of the University of Bonn, Germany (approval number: 07/21) and all subjects provided written informed consent before MRI after exclusion of MRI contraindications. All examinations were performed in accordance with relevant guidelines and regulations.

### MR examination

All study examinations were performed on a 1.5T MRI scanner (Ingenia, Philips Healthcare, Best, The Netherlands). For non-contrast MRL a sagittal heavily T2-weighted isotropic 3D spin-echo sequence (acquired voxel size: 1.2 × 1.2 × 1.2 mm, reconstructed voxel size: 0.6 × 0.6 × 0.6 mm, repetition time: 3000ms, echo time: 600ms, field-of-view: 350 × 200 × 150 mm, flip angle: 90°, acquisition time: 4:45 min) was acquired using 16-channel anterior and posterior phased array coils. Images of the entire thoracic duct from the upper abdomen to the neck were acquired with the volunteers in supine position.

Non-contrast MRL was acquired on three separate examination days in all subjects after an overnight fast of at least 8 h (baseline examination) and hourly for 6 h after ingestion of a

1. high-fat,
2. MCT- and
3. fat-free meal, respectively.

The three meals consisted of a 450 ml smoothie with a standardized composition of 100 g banana, 200 g berries and 100 ml water. They differed only in the addition of 50 ml olive oil (high-fat meal), 50 ml MCT oil (MCT-meal) or 50 ml water (fat-free meal).

### Image analysis

**Fig. 1:**
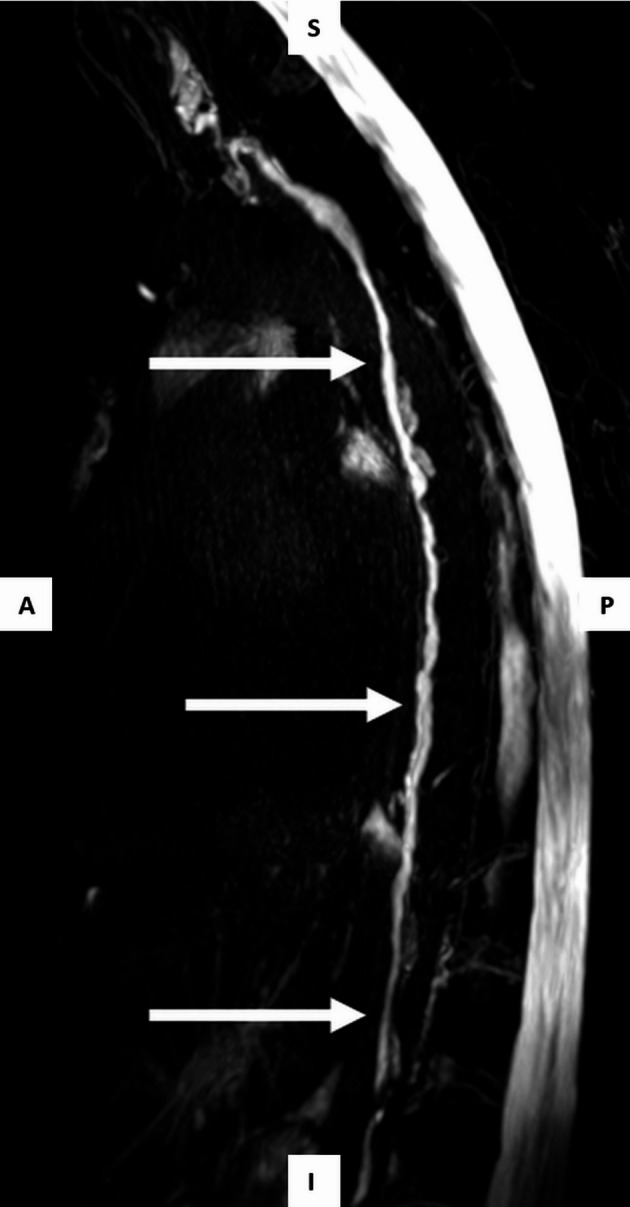
Sagittal maximum-intensity-reconstruction (MIP) of the thoracic duct (arrows). Image orientations are given as S: superior, I: inferior, A: anterior, P: posterior.

For qualitative image assessment, standardized multiplanar reconstructions with a slice thickness of 10 mm were calculated from the acquired MR-images (Fig. 1). MRLs were then evaluated independently by two readers (C.C.P, 13 years of experience; J.P., 1 year of experience). All reconstructed images were shown in random order without association to the individual volunteers to ensure blinded image review.

Overall image quality for the entire MR examination was assessed using a 5-point Likert-scale:

1. excellent (uniform contrast, no motion artifacts),
2. good (mild motion artifacts, no impairment of image interpretation),
3. moderate (motion artifacts interfering with image interpretation),
4. poor (prominent motion artifacts, diagnostic quality questionable), and.
5. non-diagnostic.

Visibility of the TD for all time-points was rated by using a 6-point score:

1. perfect (whole TD clearly visible),
2. excellent (over 90% visible),
3. good (over 75% visible),
4. moderate (over 50% visible),
5. poor (under 50% visible) and.
6. not visible.

Visibility scores were rated for the entire TD and separately for three TD-segments:

- the abdominal TD-segment below the diaphragm,
- the lower thoracic TD-segment between the diaphragm and the outflow of the pulmonary arteries,
- and upper thoracic TD-segment above the outflow of the pulmonary arteries.

Individual anatomical variations of the course of the TD were documented as

- none,
- double TD,
- partial TD-duplication, or
- plexiform TD.

These variations were evaluated along with their locations affecting either the entire TD or any of the segments.

**Fig. 2:**
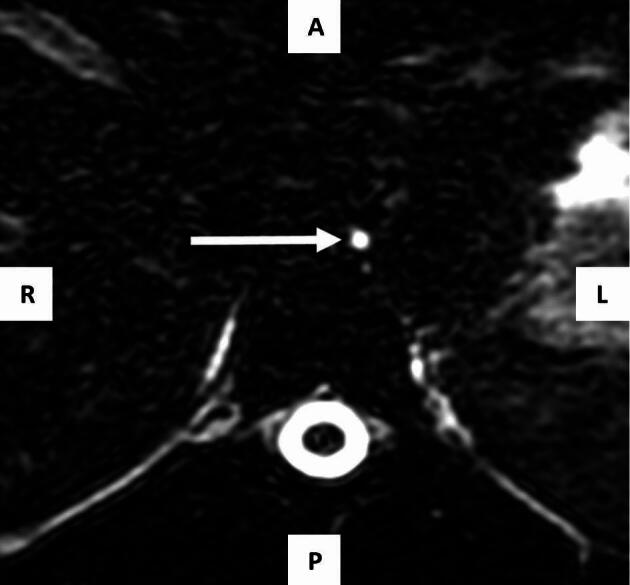
Example axial reconstruction perpendicular to the long axis of the thoracic duct (arrow) for diameter measurements. Image orientations are given as R: right, L: left, A: anterior, P: posterior.

For quantitative analysis, axial images with a slice thickness of 1 mm were reconstructed perpendicular to the long axis of the TD in consensus of both readers at the same anatomical location in the three TD-segments for all time points (Fig. 2). Measurement planes were selected at comparable anatomical levels within each thoracic duct segment where a single thoracic duct channel was identifiable across all serial examinations. Although partial duplications or short plexiform portions were documented, these anatomical variants did not involve the predefined measurement locations in a way that required selection between competing branches. The reconstructed axial images were than used by both readers independently for measuring the maximum diameter of the TD. Diameter measurements were performed using a custom-made software tool applying a full-width-at-half-maximum approach for edge-based lumen delineation. This approach was chosen to reduce the influence of partial-volume effects and subjective window-level adjustment on small-vessel diameter measurements and has previously been used for standardized vessel lumen assessment in MR angiographic data^27^.

### Statistical analysis

Statistical analysis was conducted using SPSS (version 29.0, IBM, Armonk, NY, USA). Normal distribution of the data was assessed using Q-Q plots. Values are expressed as mean ± standard deviation (SD). Overall visibility of the entire TD at baseline and at six hours after the respective meal was compared using a paired t-test for the three meals. Additionally, visibility scores of TD-segments and TD-Diameter at different time-points were compared with a one-way repeated measures analysis of variance (ANOVA).

Quantitative measurements of TD-diameters at the three different examination days were compared with a paired t-test for differences between the baseline values as well as for differences between the respective baseline and the value after 6 h. Diameters changes over time after ingestion of the MCT- and fat-rich meal were additionally assessed in comparison to the fat-free meal using a linear mixed model analysis. Interreader reliability was evaluated using the intraclass-correlation coefficient. A p-value of < 0.05 was considered as statistically significant.

## Results

Twenty-two healthy volunteers (6 male, 16 female; mean age 29.7 ± 14.3, range 19–68 years) were included into this prospective study and underwent 154 successful MRI-scans. The oral nutritional intake was well tolerated by all volunteers after the overnight fast without adverse reactions.

### Qualitative image evaluation

Overall image quality was rated as good to excellent in all examinations (mean score 1.4 ± 0.7) with motion artifacts as absent to minimal. Anatomical variations were observed in 9 cases. These included a partial duplication of the thoracic duct (abdominal TD *n* = 3, lower TD *n* = 1, upper TD *n* = 3) and short sections of a plexiform TD instead of a single TD (lower TD *n* = 2).

Visualization of the thoracic duct at baseline was slightly better on the day of the MCT-meal compared to the two other examination days for the abdominal TD-segment (MCT: 3.1 vs. high-fat: 3.3 and non-fat: 3.3, *p* = 0.035). Visibility scores did not differ between the three examination days for the lower thoracic (MCT: 2.5 vs. high-fat: 2.9 and non-fat: 2.6, *p* = 0.052) and the upper thoracic TD-segment (MCT: 2.6 vs. high-fat: 2.7 and non-fat: 2.8, *p* = 0.164).

**Fig. 3:**
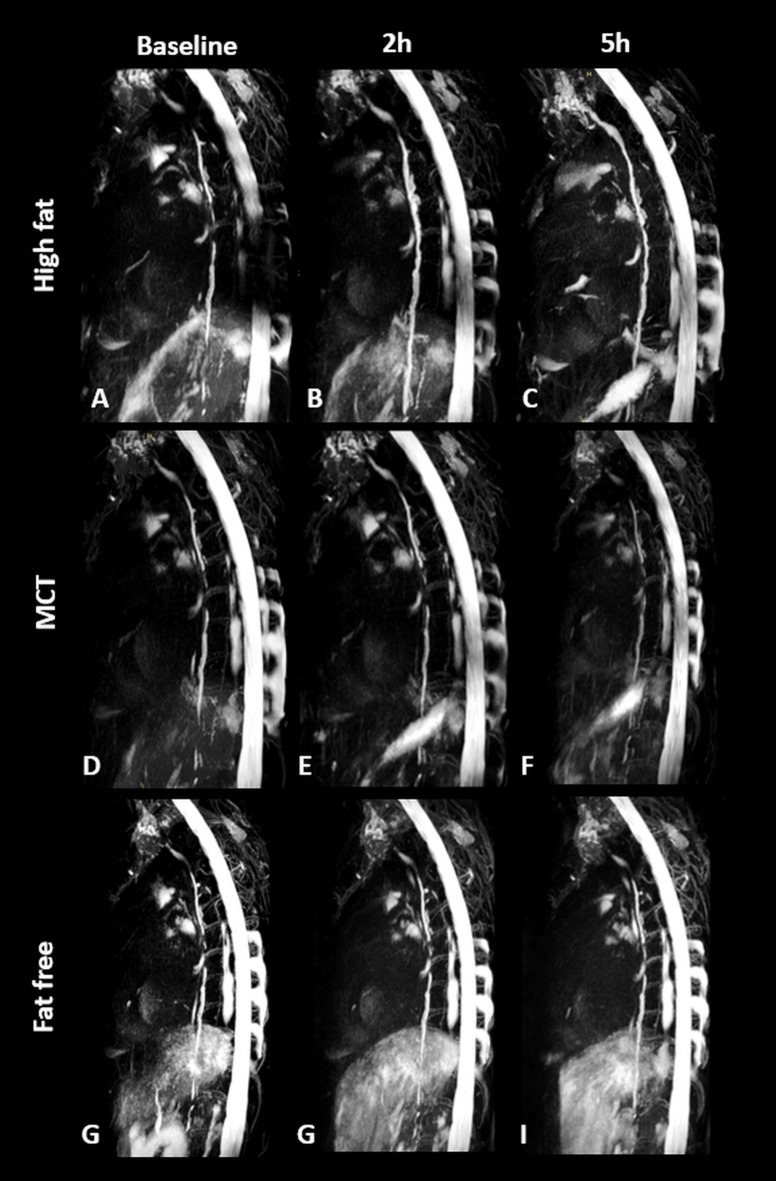
Sagittal MIPs of non-contrast MRL at baseline as well as 2 and 5 h after the high-fat meal (A-C), the MCT-meal (D-F) and the fat-free meal (G-I). Notice that there is no systematic change (fat free) or even deterioration of TD visibility (MCT) while after the high-fat meal visibility and diameter of the TD increases. Image orientations as given in Fig. 1.

**Fig. 4:**
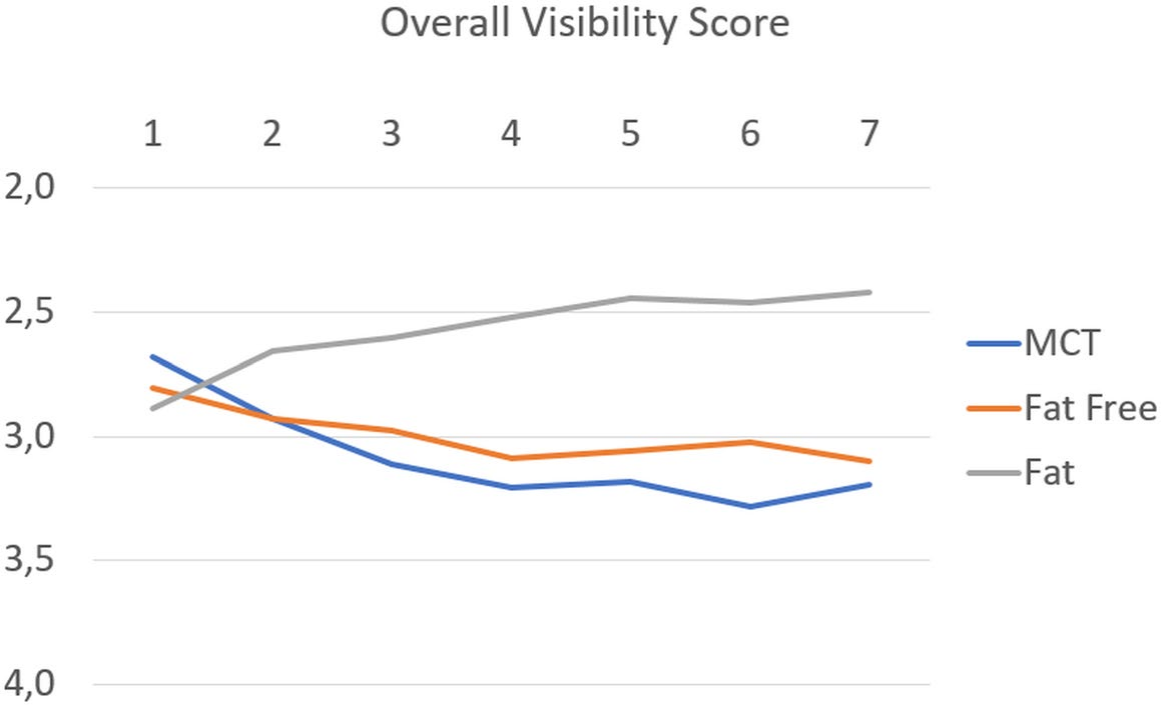
Mean overall visibility scores of the entire TD at 1: baseline and 2–7: hourly after a fat-free, MCT- and fat-rich meal over 6 h.

The overall visibility score of the whole TD improved at the postprandial examination 6 h after ingestion of the high-fat meal (2.9 ± 0.9 vs. 2.4 ± 0.8, *p* < 0.001), while it slightly deteriorated 6 h after ingestion of an MCT-meal (2.7 ± 0.9 vs. 3.2 ± 1.0, *p* < 0.001) and non-fatty meal (2.8 ± 0.9 vs. 3.1 ± 1.0, *p* = 0.006) (Figs. 3 and 4). Analysis of the individual segments of the TD showed significant improvement of TD-visibility after the high-fat meal in the abdominal and lower thoracic, but only a tread towards improved visualization in the upper thoracic segment without reaching statistical significance. After the MCT-meal visualization scores showed a trend towards (lower thorax) or even significant worsening of visualization. After the fat-free meal no changes of visualization scores were observed. With a mean visibility score of 2.4 ± 1.0 for the high-fat meal, 2.9 ± 1.0 for the MCT-meal and 2.8 ± 1.0 for the fat-free meal, the visibility score of the lower thoracic portion of the TD was highest compared to the other segments, but without reaching statistical significance (see Table 1 for details).

**Table 1:** Mean visibility scores of the anatomical segments of the thoracic duct overall, at baseline (i.e. after overnight fasting) and hourly after a fat-free, MCT- and fat-rich meal. Visibility score: 1: perfect, 6: not visible. p-values derived from one-way repeated measures ANOVA. MCT: medium chain triglyceride.

	Thoracic duct visibility score								
	Overall	Baseline	1 h	2 h	3 h	4 h	5 h	6 h	*p*-value
Fat-free meal									
Abdomen	3.3 ± 0.9	3.3 ± 0.9	3.3 ± 0.9	3.4 ± 1.0	3.4 ± 0.9	3.3 ± 1.0	3.3 ± 1.0	3.3 ± 1.0	0.749
Lower thoracic	2.8 ± 1.0	2.6 ± 0.8	2.8 ± 1.1	2.8 ± 1.0	2.9 ± 1.1	2.8 ± 1.0	2.9 ± 1.0	2.9 ± 1.1	0.138
Upper thoracic	3.0 ± 0.8	3.0 ± 1.1	3.0 ± 1.1	3.0 ± 0.7	3.1 ± 0.7	3.1 ± 0.9	3.0 ± 0.8	3.0 ± 0.9	0.184
MCT meal									
Abdomen	3.4 ± 0.9	3.1 ± 0.9	3.3 ± 0.9	3.5 ± 1.0	3.5 ± 1.0	3.5 ± 0.9	3.5 ± 0.9	3.5 ± 1.0	**0.037**
Lower thoracic	2.9 ± 1.0	2.5 ± 0.9	2.7 ± 0.9	2.9 ± 0.9	3.0 ± 0.9	3.0 ± 1.1	3.0 ± 1.1	2.9 ± 0.9	0.141
Upper thoracic	3.0 ± 0.9	2.6 ± 0.7	2.7 ± 0.6	3.0 ± 0.8	3.2 ± 1.0	3.1 ± 1.0	3.4 ± 1.1	3.0 ± 0.8	**0.015**
High-fat meal									
Abdomen	2.9 ± 0.9	3.3 ± 0.8	3.0 ± 1.0	2.9 ± 0.9	2.9 ± 0.8	2.7 ± 0.8	2.7 ± 0.8	2.7 ± 0.8	**0.002**
Lower thoracic	2.4 ± 1.0	2.9 ± 1.0	2.5 ± 1.1	2.4 ± 1.0	2.4 ± 1.0	2.2 ± 0.9	2.2 ± 0.9	2.2 ± 0.9	**0.021**
Upper thoracic	2.5 ± 0.7	2.7 ± 0.6	2.5 ± 0.7	2.5 ± 0.9	2.5 ± 0.7	2.4 ± 0.7	2.4 ± 0.7	2.4 ± 0.7	0.206

### Quantitative image evaluation

**Fig. 5:**
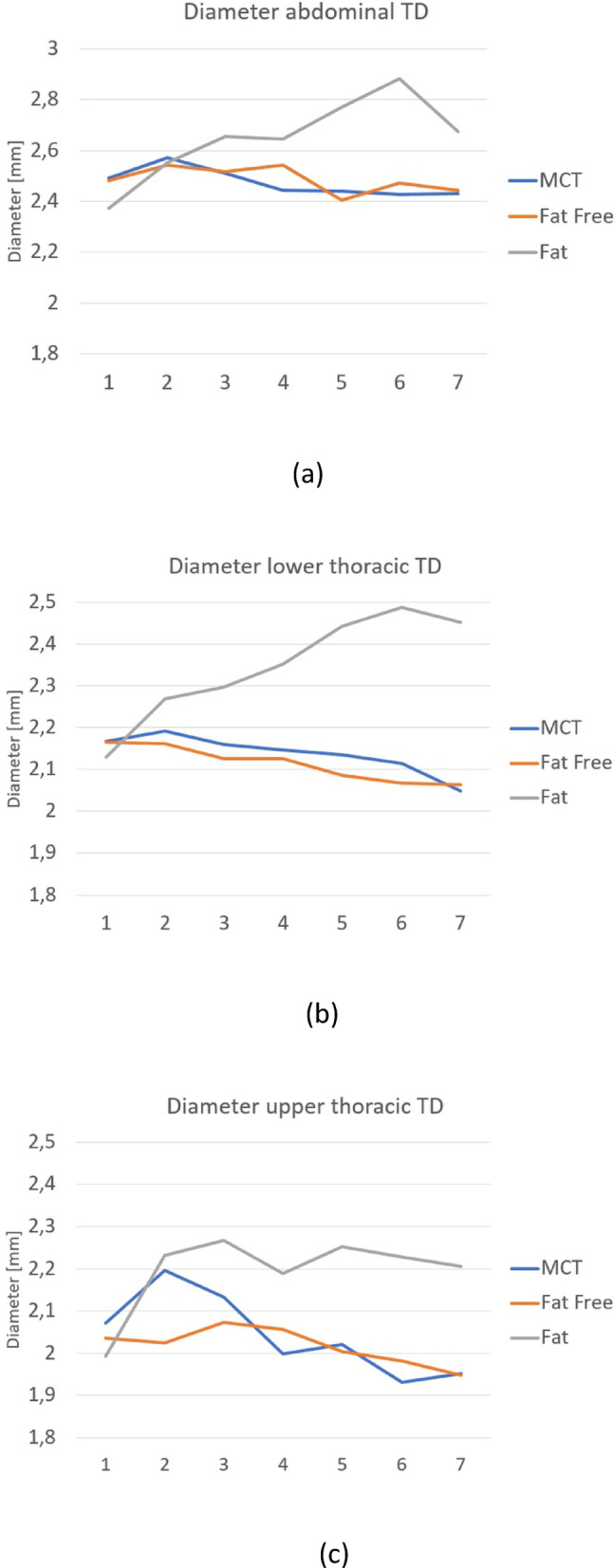
Mean diameters of the anatomical sections of the TD (**A**: abdominal segment, **B**: lower thoracic segment **C**: upper thoracic segment) at the seven time-points; 1: baseline and 2–7: hourly after a fat-free, MCT- and fat-rich meal over 6 h.

Diameter measurements of the TD were possible at the same anatomical location between the different time points in all cases without artifacts obscuring the TD. Described anatomical variations did not interfere with diameter measurements in any of the cases as there was a single duct in each of the TD-segments for evaluation. Detailed measurement results are given in Table 2 and Fig. 5.

**Table 2:** Mean thoracic duct diameters measured for each anatomical section of the thoracic duct at baseline (i.e. after overnight fasting) and hourly after a fat-free, MCT- and fat-rich meal. MCT: medium chain triglyceride.

	Thoracic duct diameter [mm]						
	Baseline	1 h	2 h	3 h	4 h	5 h	6 h
Fat-free meal							
Abdomen	2.48 ± 0.7	2.54 ± 0.9 (+ 2.5%)	2.52 ± 0.9 (+ 1.4%)	2.54 ± 0.9 (+ 2.5%)	2.40 ± 0.8 (− 3.1%)	2.47 ± 0.8 (− 0.4%)	2.44 ± 0.9 (− 1.5%)
Lower thoracic	2.17 ± 0.4	2.16 ± 0.5 (− 0.2%)	2.13 ± 0.4 (− 1.8%)	2.13 ± 0.5 (− 1.8%)	2.09 ± 0.4 (− 3.7%)	2.07 ± 0.4 (− 4.6%)	2.06 ± 0.4 (− 4.7%)
Upper thoracic	2.04 ± 0.2	2.02 ± 0.2 (− 0.6%)	2.07 ± 0.2 (+ 1.8%)	2.06 ± 0.3 (+ 0.9%)	2.00 ± 0.2 (− 1.6%)	1.98 ± 0.2 (− 2.7%)	1.95 ± 0.2 (− 4.3%)
MCT meal							
Abdomen	2.49 ± 0.7	2.57 ± 0.8 (+ 3.1%)	2.51 ± 0.9 (+ 0.8%)	2.44 ± 0.9 (− 2.0%)	2.44 ± 0.8 (− 2.1%)	2.43 ± 0.7 (− 2.6%)	2.43 ± 0.8 (− 2,6%)
Lower thoracic	2.17 ± 0.4	2.19 ± 0.5 (+ 1.1%)	2.16 ± 0.5 (− 0.3%)	2.15 ± 0.4 (− 0.9%)	2.14 ± 0.4 (− 1.4%)	2.12 ± 0.4 (− 2.4%)	2.05 ± 0.3 (− 5.4%)
Upper thoracic	2.07 ± 0.3	2.2 ± 0.4 (+ 6.0%)	2.13 ± 0.3 (+ 3.0%)	2.00 ± 0.4 (− 3.5%)	2.02 ± 0.2 (− 2.4%)	1.93 ± 0.2 (− 6.7%)	1.95 ± 0.2 (− 5.7%)
High-fat meal							
Abdomen	2.37 ± 0.6	2.55 ± 0.7 (+ 7,6%)	2.66 ± 0.8 (+ 12.0%)	2.64 ± 0.8 (+ 11.5%)	2.77 ± 1.0 (+ 16.9%)	2.88 ± 1.0 (+ 21.5%)	2.67 ± 0.9 (+ 12.7%)
Lower thoracic	2.13 ± 0.4	2.27 ± 0.6 (+ 6.5%)	2.30 ± 0.5 (+ 7.8%)	2.35 ± 0.5 (+ 10.4%)	2.44 ± 0.6 (+ 14.6%)	2.49 ± 0.5 (+ 16.8%)	2.45 ± 0.5 (15.1%)
Upper thoracic	1.99 ± 0.3	2.23 ± 0.5 (+ 12.0%)	2.27 ± 0.5 (+ 13.8%)	2.19 ± 0.4 (+ 9.8%)	2.25 ± 0.4 (13.1%)	2.23 ± 0.4 (+ 11.8%)	2.20 ± 0.5 (10.7%)

There was a tendency towards lower baseline TD-diameters in the abdominal, lower and upper part of the thoracic duct on the day of the high-fat meal (mean diameters overall 2.17 ± 0.5 mm) compared to the MCT (2.24 ± 0.5 mm) and fat-free meal (2.23 ± 0.5 mm) without reaching statistical significance (*p* > 0.05).

When comparing the values between baseline and the maximum changes after the meals, the TD-diameter increased significantly after the high-fat meal in the abdominal (2.37 ± 0.6 mm at baseline vs. 2.88 ± 1.0 mm at 5 h, + 21.5%, *p* < 0.001), the lower thoracic (2.13 ± 0.4 mm at baseline vs. 2.49 ± 0.5 mm at 5 h, + 16.8%, *p* < 0.001) and the upper thoracic segment (1.99 ± 0.3 mm at baseline vs. 2.27 ± 0.5 mm at 2 h, + 13.8%, *p* = 0.001). In contrast, no significant increase in TD-diameters over time was observed for the fat-free and MCT-meals. There was even a trend towards decreasing diameters after both meals between 4 and 6 h after ingestion.

Due to the observed variation in the baseline measurements that might affect this simple comparative testing of postprandial diameters to the one baseline value, a linear mixed model taking changes over time into account was additionally used for further analysis. Mixed model analysis confirmed that there was no statistically significant change in TD-diameter after ingestion of the fat-free as well as the MCT-meal within the observation period. In contrast, the increase in mean diameters of the TD after the high-fat meal was statistically significant (*p* < 0.001).

Intraclass-correlation coefficient calculation demonstrated good to excellent agreement between the readers (abdominal: 0.812, [95%-CI 0.722; 0.875], lower thoracic: 0.901 [0.827; 0.978], upper thoracic: 0.856 [0.789; 0.903).

## Discussion

Magnetic resonance lymphangiography (MRL) has been increasingly employed in recent years as new interventional and medical treatment options for lymphatic diseases have become available^16,17,28^. Non-contrast MRL offers several advantages over contrast-enhanced examinations: The employed heavily T2-weighted 3D-sequences are technically easier to implement into clinical routine imaging than logistically more demanding contrast-enhanced techniques. Furthermore, non-contrast MRL is completely non-invasive and is therefore more appealing especially in pediatric patients with suspected lymphatic diseases. Finally, non-contrast MRL is useful for follow-up examinations forgoing the need for repeated contrast-application^24^. However, as non-contrast MRL relies solely on the morphological depiction of larger lymphatic vessels using a single, time-consuming 3D-sequence, optimizing the chance of obtaining an adequate image is important. Apart from technical considerations, influencing the potential visibility of central lymphatics by exploiting physiological processes is an option to improve image quality.

In parallel, the field of lymphatic imaging has continued to evolve rapidly. Especially intranodal lymphangiography, dynamic contrast-enhanced MR lymphangiography, and lymphatic interventions have broadened the spectrum for diagnosing and treating congenital and acquired lymphatic disorders^29,30^. In patients with congenital heart disease, non-contrast T2-weighted lymphatic MRI has also gained increasing relevance for screening and risk stratification, with recent studies showing that lymphatic abnormalities may already be present before Glenn operation and may progress during the course of Fontan completion^31,32^. Against this background, improving the robustness and physiological interpretability of non-contrast thoracic duct imaging remains clinically relevant.

The present study examined this concept of improving TD-visibility and possible TD-diameter changes through ingestion of meals with different fat contents. The main findings were: First, visibility especially of the lower TD-sections is improved after a high-fat meal, while both after an MCT- and a fat-free meal there was no improvement or even a deterioration in TD-visibility. Second, quantitative TD-diameter values increased significantly after ingestion of a high-fat meal, while there was no significant change after an MCT- and fat-free meal. Third, dynamic diameter measurements revealed that there was no significant difference between the fat-free and the MCT-meal concerning qualitative visibility scores as well as quantitative diameter measurements.

A small number of previous studies have explored the effects a high-fat meal several hours before imaging on TD-visibility^4,5,11^. In general, prior studies found an improvement of TD-visibility. The results of our study are in line with these prior findings, demonstrating in the largest cohort of healthy individuals so far that there is a significant improvement in TD-visibility on non-contrast MRL after a high-fat meal compared to a fasting state. This improvement was most pronounced in the abdominal and lower thoracic TD-segment. This may to some degree be due to a generally more difficult visualization of the upper TD because of pulsation of the adjoining aortic arch. The improvement of TD-visibility may reflect an increase in TD-diameter secondary to an anticipated rise in lymph production during intestinal absorption of long-chain triglycerides. We observed an increase in TD-diameter of up to 21% compared to the baseline (2.4 mm vs. 2.9 mm) which is comparable to an increase from 3.5 ± 0.6 mm to 3.9 ± 1.1 mm reported in one single previous study^11^. However, the mechanism of fat-resorption via the central lymphatic system is so far not fully understood^4^. It is known that triglyceride content of chyle within the central lymphatic system increases after ingestion of fatty meals^33^ and that this increase can be avoided by a fat-free meal^1^. Additionally, it has been demonstrated previously that changes in lymph production/lymph flow in the TD leads to alterations of the TD-diameter. In patients with liver cirrhosis the diameter of the TD and/or the cisterna chyli is significantly increased because of elevated lymph production due to portal hypertension. Conversely, creation of a transjugular intrahepatic portosystemic shunt (TIPS) in the treatment of decompensated cirrhosis leads to a significant decrease in the caliber of central lymphatics^34^. Nevertheless, lymph flow, chylomicron transport, and triglyceride concentrations within the thoracic duct were not directly measured in the present study. The observed morphological changes should therefore be interpreted as indirect imaging correlates consistent with the expected postprandial lymphatic response rather than as direct proof of increased lymphatic resorption.

Interestingly, the maximum effect of visibility improvement varies considerably between studies. In contrast to two previous studies^5,11^ we did not observe a peak of visibility at three hours after the meal, but considerably later at five to six hours. This is more in line with a study by Nomura et al.^4^. In contrast to our study, previous works administered 50–60 g of oral fat in the form of ice cream or a drink of full-fat cream prior to non-contrast MRL^4,5^, whereas participants in our study received a fruit smoothy with added olive oil. Although the amount of fat was comparable with previous studies, differences in digestion and resorption between cream and olive-oil may therefore partly explain the difference in the time to the peak effect. We opted for this form of oral fat due to the following considerations: We intended to (1) keep the volume of the meal on all days constant with only a variation in the fat-content, to (2) be able to substitute the olive-oil (long-chain fatty acids) with MCT-oil without changing the rest of the composition of the meal and (3) to exclude all animal-derived products to enable the inclusion of also vegan participants.

The findings of this study are clinically relevant because non-contrast MRL is an increasingly employed tool in the screening for lymphatic abnormalities, especially in patients with congenital heart disease^35^. In patients with univentricular heart disease in particular, the grading of the extent of lymphatic abnormalities in the thorax and especially the mediastinum is closely associated with patient outcome and complications rates after Fontan-surgery with worse outcomes in higher grades of abnormalities. Improving the image quality of non-contrast MRL by ingestion of a fatty meal several hours prior to the examination therefore seems to be a worthwhile strategy. It has to be kept in mind, however, that especially in pediatric patients, sedation or general anesthesia may be mandatory for the MR-examination. A high-fat meal and the required time interval between this meal and the examination must be discussed with the anesthesiology department well in advance.

So far, the effect of an MCT-meal on visibility and maximum diameter of the TD on MRI has not been investigated. However, MCT-diet is an integral part of the standard conservative treatment of central chylous leakages^17^ and has been used in this indication for decades^36,37^. Typical causes of chylous effusions include traumatic (most frequently iatrogenic) injury of central lymphatic vessels^2,38,39^ as well as a large spectrum of non-traumatic pathologies primarily or secondarily affecting the central lymphatic system^13,40^. The resulting chylous effusions are associated with considerable morbidity and mortality due to nutritional depletion and immunodeficiency^1,5,36,37,40,41^. Patients with chylous leakages who receive an MCT-diet free from long-chain fatty acids usually demonstrate reduced leakage volumes and decreasing triglyceride values in the drained fluid^36^. These effects of an MCT-diet are commonly attributed to a decreased lymph flow in the TD as the absorption of short- and medium-chain fatty acids does not lead to increased lymph production in the bowel thereby effectively decreasing central lymph flow^2,13–15^. The findings of the present study are consistent with this physiological concept. In contrast to the high-fat meal, there was no improvement in TD-visibility or increase in TD-diameter after the MCT-meal compared to the fasting state. Consecutive measurements after the MCT-meal rather showed comparable results with the fat-free meal.

There are several noteworthy limitations to the present study that have to be discussed. First, the study included only healthy adult subjects. The observed effects may differ in children or elderly people as well as in patients with lymphatic diseases. In addition, the study cohort showed an unequal sex distribution, with a predominance of female volunteers. Potential sex-related differences in thoracic duct anatomy, lymphatic flow, or postprandial lymphatic response could therefore not be assessed. The findings should consequently be interpreted as group-level observations in a healthy adult cohort and may not be fully generalizable to more balanced populations or to specific patient groups. Secondly, the three meals were chosen for optimal comparability, but do not represent a wholesome diet. Other kinds of nutrition may have different effects on central lymphatics. Thirdly, despite using an optimized non-contrast MRL sequence, there were still some motion artefacts from the heart or breathing possibly affecting manual measurements of TD-diameters. New imaging approaches with accelerated imaging may in future make simultaneous breathing-guidance and cardiac-triggering possible at acceptable scan times. However, using this optimized, high-resolution T2w 3D-sequence was associated with an overall good to excellent TD-visualization already at baseline. It is arguable, that improvement of TD-visibility may be more pronounced when employing a faster and less resolved sequence. This, however, was not the aim of the present study. Another technical limitation in this respect concerns the spatial resolution of the sequence in relation to the small absolute diameter changes observed in some thoracic duct segments. Although an optimized isotropic 3D sequence was used and diameter measurements were performed with a full-width-at-half-maximum approach to reduce the influence of edge blurring, some of the smaller diameter changes, particularly in the upper thoracic segment, were close to the spatial resolution limits of the sequence. Therefore, these smaller changes should be interpreted primarily as group-level observations rather than precise individual-level diameter measurements. Fourth, we did not investigate changes of the cisterna chyli, the mesenteric or lumbar trunks in this study, because the anatomy of these structures is highly variable^41,42^. Besides the normal variant of a single TD, complete or partial duplications as well as plexiform TDs can also be observed frequently^41^. These anatomical variations obviously would make reliable and comparable measurements between different volunteers or patients impossible. As was to be expected, we observed a number of partial duplications of the TD in our cohort. However, no volunteer had to be excluded from analysis as a single TD was present in comparable anatomical locations in all cases. A further limitation relates to the statistical handling of the qualitative visibility scores. Visibility was assessed using an ordinal score and was analyzed as a supportive imaging parameter rather than as the main quantitative endpoint of the study. Therefore, the results of the qualitative visibility analysis should be interpreted with appropriate caution. The primary statistical inference was based on quantitative thoracic duct diameter measurements. Lastly, as in previous studies, we observed large intra- and inter-individual variations of TD-visualization and diameter. This is due to the fact that lymph flow can be affected by several extrinsic and intrinsic factors^43–46^. We therefore chose to include a mixed model analysis to assess quantitative changes in diameters in order not only to compare single measurements, but also to take dynamic changes over time into account. This model accounts for the repeated-measures design and for changes over time relative to the respective same-day baseline, thereby addressing potential baseline differences between examination days. One has to keep in mind, however, that the described observations are a group effect, not necessarily visible in every single patient.

In conclusion, non-invasive assessment of the thoracic duct by non-contrast MR lymphangiography using a heavily T2-weighted 3D-sequence can be improved by a high-fat meal five to six hours prior to imaging. Using this approach, visibility of the TD improves especially in the abdominal and lower thoracic segment. The observed improvement in TD-visibility may be related to an increase in TD-diameter in the context of anticipated lymphatic resorption of long-chain triglycerides. Conversely, comparable effects are not present after a fat-free meal. Additionally, visibility and diameter of the TD do not change significantly after an MCT-meal supporting the use of an MCT-diet in conservative treatment of central lymphatic leakages.

## Data Availability

The data that support the findings of this study are not publically available due to data protection regulations but are available from the corresponding author upon reasonable request.

## References

[1] Sriram, K., Meguid, R. & Meguid, M. Nutritional support in adults with chyle leaks. *Nutrition* **32**, 281–286 (2016). 26472113 10.1016/j.nut.2015.08.002

[2] Nair, S. K., Petko, M. & Hayward, M. P. Aetiology and management of chylothorax in adults. *Eur. J. Cardiothorac. Surg.* **32**(2), 362–369 (2007). 17580118 10.1016/j.ejcts.2007.04.024

[3] Phang, K., Bowman, M., Phillips, A. & Windsor, J. Review of thoracic duct anatomical variations and clinical implications. *Clin. Anat.* **27**(4), 637–644 (2014). 24302465 10.1002/ca.22337

[4] Nomura, T. et al. Magnetic resonance thoracic ductography assessment of serial changes in the thoracic duct after the intake of a fatty meal. *J. Anat.* **232**(3), 509–514 (2018). 29226328 10.1111/joa.12761 PMC5807931

[5] Chen, S. et al. Non-enhanced MR lymphography of the thoracic duct: improved visualization following ingestion of a high fat meal–initial experience. *Clin. Physiol. Funct. Imaging*. **37**(6), 730–733 (2017). 27555355 10.1111/cpf.12366

[6] Kassis, T. et al. Postprandial lymphatic pump function after a high-fat meal: A characterization of contractility, flow, and viscosity. *Am. J. Physiol. Gastrointest. Liver Physiol.* **310**(10), G776-89 (2016). 26968208 10.1152/ajpgi.00318.2015 PMC4888550

[7] Bloom, B., Chaikoff, I. L. & Reinhardt, W. O. Intestinal lymph as pathway for transport of absorbed fatty acids of different chain lengths. *Am. J. Physiol-Leg Content* **166**(2), 451–455 (1951). 10.1152/ajplegacy.1951.166.2.451 14857202

[8] Dixon, J. B. Lymphatic lipid transport: sewer or subway? *Trends Endocrinol. Metab.* **21**(8), 480–487 (2010). 20541951 10.1016/j.tem.2010.04.003 PMC2914116

[9] Randolph, G. J. & Miller, N. E. Lymphatic transport of high-density lipoproteins and chylomicrons. *J. Clin. Invest.* **124**(3), 929–935 (2014). 24590278 10.1172/JCI71610 PMC3934183

[10] Iqbal, J. & Hussain, M. M. Intestinal lipid absorption. *Endocrinol. Metab.* **296** (2009). 10.1152/ajpendo.90899.2008 PMC2692399 19158321

[11] Hanser, A. et al. T2-weighted high-resolution isotropic magnetic resonance lymphangiography of the thoracic and abdominal lymphatic vessels with and without previous high-fat meal. *Acad. Radiol.* **28**, S218–S224 (2021). 33183951 10.1016/j.acra.2020.10.008

[12] Tso, P., Pitts, V. & Granger, D. N. Role of lymph flow in intestinal chylomicron transport. *Am. J. Physiol-Gastrointest Liver Physiol.* **249**(1), G21–G28 (1985). 10.1152/ajpgi.1985.249.1.G21 4014464

[13] Riley, L. E. & Ataya, A. Clinical approach and review of causes of a chylothorax. *Respir. Med.* **157**, 7–13 (2019). 31454675 10.1016/j.rmed.2019.08.014

[14] Kim, E. Y. et al. Anatomic and functional evaluation of central lymphatics with noninvasive magnetic resonance lymphangiography. *Medicine* **95**(12), e3109 (2016). 27015184 10.1097/MD.0000000000003109 PMC4998379

[15] Alvarez, J., Kalache, K. & Grauel, E. Management of spontaneous congenital chylothorax: Oral medium-chain triglycerides versus total parenteral nutrition. *Am. J. Perinatol.* **16**(08), 0415–20 (1999). 10.1055/s-1999-6816 10772201

[16] Pieper, C. C. et al. Back to the future: Lipiodol in lymphography-from diagnostics to theranostics. *Invest. Radiol.* **54**(9), 600–615 (2019). 31283538 10.1097/RLI.0000000000000578

[17] Pieper, C. C. Back to the future II-A comprehensive update on the rapidly evolving field of lymphatic imaging and interventions. *Invest. Radiol.* **58**(8), 610–640 (2023). 37058335 10.1097/RLI.0000000000000966

[18] Pieper, C. C. Nodal and pedal MR lymphangiography of the central lymphatic system: Techniques and applications. *Semin. Intervent. Radiol.* **37**(3), 250–262 (2020). 32773950 10.1055/s-0040-1713442 PMC7394572

[19] Jara, H. et al. MR hydrography: theory and practice of static fluid imaging. *Am. J. Roentgenol.* **170**(4), 873–882 (1998). 9530026 10.2214/ajr.170.4.9530026

[20] Hayashi, S. & Miyazaki, M. Thoracic duct: Visualization at nonenhanced MR lymphography—initial experience. *Radiology* **212**(2), 598–600 (1999). 10429724 10.1148/radiology.212.2.r99au23598

[21] Takahashi, H. et al. Clinical feasibility of noncontrast-enhanced magnetic resonance lymphography of the thoracic duct. *Chest* **124**(6), 2136–2142 (2003). 14665492 10.1378/chest.124.6.2136

[22] Matsushima, S., Ichiba, N., Hayashi, D. & Fukuda, K. Nonenhanced magnetic resonance lymphoductography: Visualization of lymphatic system of the trunk on 3-dimensional heavily T2-weighted image with 2-dimensional prospective acquisition and correction. *J. Comput. Assist. Tomogr* **31**(2), 299 (2007). 17414769 10.1097/01.rct.0000236415.97642.58

[23] Okuda, I., Udagawa, H., Hirata, K. & Nakajima, Y. Depiction of the thoracic duct by magnetic resonance imaging: comparison between magnetic resonance imaging and the anatomical literature. *Jpn J. Radiol.* **29**(1), 39–45 (2011). 21264660 10.1007/s11604-010-0515-0

[24] Yu, D. X., Ma, X. X., Wang, Q., Zhang, Y. & Li, C. F. Morphological changes of the thoracic duct and accessory lymphatic channels in patients with chylothorax: detection with unenhanced magnetic resonance imaging. *Eur. Radiol.* **23**(3), 702–711 (2013). 22976916 10.1007/s00330-012-2642-8

[25] Liu, N. & Zhang, Y. Magnetic resonance lymphangiography for the study of lymphatic system in lymphedema. *J. Reconstr. Microsurg* **32**(01), 066–071 (2014). 10.1055/s-0034-1384213 25025507

[26] Arrivé, L. et al. Noncontrast magnetic resonance lymphography. *J. Reconstr. Microsurg* **32**(01), 080–086 (2015). 10.1055/s-0035-1549133 25826439

[27] Merkx, M. A. G. et al. Accuracy and precision of vessel area assessment: Manual versus automatic lumen delineation based on full-width at half-maximum. *J. Magn. Reson. Imaging*. **36**(5), 1186–1193 (2012). 22826150 10.1002/jmri.23752

[28] Pieper, C. C. et al. MR lymphangiography of lymphatic abnormalities in children and adults with Noonan syndrome. *Sci. Rep.* **12**(1), 11164 (2022). 35778409 10.1038/s41598-022-13806-w PMC9249771

[29] Benjamin, J. et al. Imaging and interventions for lymphatic and lymphatic-related disorders. *Radiology* **307**(3), e220231 (2023). 36943078 10.1148/radiol.220231

[30] Negm, A. S. et al. MR lymphangiography in lymphatic disorders: Clinical applications, institutional experience, and practice development. *Radiographics* **44**(2), e230075 (2024). 38271257 10.1148/rg.230075

[31] Kelly, B. et al. Sequential MRI evaluation of lymphatic abnormalities over the course of Fontan completion. *Radiol. Cardiothorac. Imaging* **6**(3), e230315 (2024). 38814187 10.1148/ryct.230315 PMC11211943

[32] Kristensen, R. et al. Lymphatic abnormalities on magnetic resonance imaging in single-ventricle congenital heart defects before Glenn operation. *J. Am. Heart Assoc.* **12**(12), e029376 (2023). 37318013 10.1161/JAHA.123.029376 PMC10356053

[33] Robinson, C. L. N. The management of chylothorax. *Ann. Thorac. Surg.* **39**, 90–95 (1985). 3966842 10.1016/s0003-4975(10)62531-3

[34] Pieper, C. C. et al. Impact of transjugular intrahepatic portosystemic shunt creation on the central lymphatic system in liver cirrhosis. *Sci. Rep.* **11**(1), 7065 (2021). 33782430 10.1038/s41598-021-86006-7 PMC8007746

[35] Biko, D. M. et al. MRI evaluation of lymphatic abnormalities in the neck and thorax after Fontan surgery: Relationship with outcome. *Radiology* **291**(3), 774–780 (2019). 30938628 10.1148/radiol.2019180877 PMC6542623

[36] Schild, H. H., Strassburg, C. P., Welz, A. & Kalff, J. Treatment options in patients with chylothorax. *Dtsch. Arztebl. Int.* **110**(48), 819–26 (2013). 24333368 10.3238/arztebl.2013.0819 PMC3865492

[37] Schild, H. H. & Pieper, C. Chylothorax: eine Übersicht über aktuelle therapeutische Möglichkeiten Chylothorax: Current Therapeutic Options. *Zentralbl Chir.* **144**(S 01), S24–S30 (2019). 30795028 10.1055/a-0831-2649

[38] Doerr, C. H., Allen, M. S., Nichols, F. C. & Ryu, J. H. Etiology of chylothorax in 203 patients. *Mayo Clin. Proc.* **80**(7), 867–70 (2005). 16007891 10.4065/80.7.867

[39] Weijs, T. J. et al. The peri-esophageal connective tissue layers and related compartments: Visualization by histology and magnetic resonance imaging. *J. Anat.* **230**(2), 262–71 (2017). 27659172 10.1111/joa.12552 PMC5244460

[40] Valentine, V. G. & Raffin, T. A. The management of chylothorax. *Chest* **102**(2), 586–591 (1992). 1643953 10.1378/chest.102.2.586

[41] Johnson, O. W. et al. The thoracic duct: Clinical importance, anatomic variation, imaging, and embolization. *Eur. Radiol.* **26**(8), 2482–93 (2016). 26628065 10.1007/s00330-015-4112-6

[42] Erden, A., Fitoz, S., Yagmurlu, B. & Erden, I. Abdominal confluence of lymph trunks: detectability and morphology on heavily T2-weighted images. *AJR* **184**(1), 35–40 (2005). 15615947 10.2214/ajr.184.1.01840035

[43] Gashev, A. A. Lymphatic vessels: Pressure- and flow‐dependent regulatory reactions. *Ann. N. Y. Acad. Sci.* **1131**(1), 100–109 (2008). 18519963 10.1196/annals.1413.009

[44] Gashev, A. A. & Zawieja, D. C. Hydrodynamic regulation of lymphatic transport and the impact of aging. *Pathophysiology* **17**(4), 277–287 (2010). 20226639 10.1016/j.pathophys.2009.09.002 PMC5507682

[45] Scallan, J. P., Zawieja, S. D., Castorena-Gonzalez, J. A. & Davis, M. J. Lymphatic pumping: mechanics, mechanisms and malfunction. *J. Physiol.* **594**(20), 5749–5768 (2016). 27219461 10.1113/JP272088 PMC5063934

[46] Moriondo, A., Mukenge, S. & Negrini, D. Transmural pressure in rat initial subpleural lymphatics during spontaneous or mechanical ventilation. *Am. J. Physiol-Heart Circ. Physiol.* **289**(1), H263–H269 (2005). 15833809 10.1152/ajpheart.00060.2005

